# GD-StarGAN: Multi-domain image-to-image translation in garment design

**DOI:** 10.1371/journal.pone.0231719

**Published:** 2020-04-21

**Authors:** Yangyun Shen, Runnan Huang, Wenkai Huang

**Affiliations:** 1 School of Fine Art and Artistic Design, Guangzhou University, Guangzhou, China; 2 Center for Research on Leading Technology of Special Equipment, School of Mechanical and Electrical Engineering, Guangzhou University, Guangzhou, China; 3 School of Mechanical and Electrical Engineering, Guangzhou University, Guangzhou, China; Beijing University of Technology, CHINA

## Abstract

In the field of fashion design, designing garment image according to texture is actually changing the shape of texture image, and image-to-image translation based on Generative Adversarial Network (GAN) can do this well. This can help fashion designers save a lot of time and energy. GAN-based image-to-image translation has made great progress in recent years. One of the image-to-image translation models––StarGAN, has realized the function of multi-domain image-to-image translation by using only a single generator and a single discriminator. This paper details the use of StarGAN to complete the task of garment design. Users only need to input an image and a label for the garment type to generate garment images with the texture of the input image. However, it was found that the quality of the generated images is not satisfactory. Therefore, this paper introduces some improvements on the structure of the StarGAN generator and the loss function of StarGAN, and a model was obtained that can be better applied to garment design. It is called GD-StarGAN. This paper will demonstrate that GD-StarGAN is much better than StarGAN when it comes to garment design, especially in texture, by using a set of seven categories of garment datasets.

## Introduction

The process of garment design is to conceive the garment according to the requirements of the design object, draw the garment image, and then make the garment according to the drawings to complete the whole design process. On the design process, there are some tasks that require a lot of time. For example, if the same texture is to be used in different types of garments, it requires designers to design them individually to obtain the garment image, which is time-consuming and energy-consuming. However, these tasks can be completely solved with image-to-image translation technology.

Language translation is the transformation of expressions between languages, and image-to-image translation is similar; It is a transformation of different styles of images, such as shape style, color style, etc. A texture image is transformed into images of various types of garments with the same texture, which is an application of image-to-image translation. Nowadays, the emergence of Generative Adversarial Network (GAN) [1] has greatly promoted the development of image-to-image translation. Pix2Pix [2] is based on Conditional GAN(CGAN) [3], which is a model of image-to-image translation using paired dataset training. CycleGAN [4] and DiscoGAN [5], which were proposed at almost the same time, relieve dependence on the paired dataset and realize the image-to-image translation of two domains without pairing processing of the dataset. However, although the above work [2], [4], [5] can achieve excellent image-to-image translation, they are limited to two domains. When encountering multiple domains, these methods are limited and inconvenient. Using these models to achieve multi-domain image-to-image translation requires building models independently for each domain, which makes the network too large, requires a large amount of graphics processing unit memory, and may generate low-quality images.

StarGAN [6], another image-to-image translation model, has demonstrated its powerful ability in multi-domain image-to-image translation. It consists of a discriminator and a generator for translating multi-domain facial images. This feature of StarGAN is very suitable for helping fashion designers to design different types of garments with the same texture. StarGAN is used for face conversion, and it can generate high-quality images. However, the garment design based on image-to-image translation in this paper is much more difficult than face image-to-image translation. Because in the garment dataset, for the same type of garment, their texture and size may be varied, which has a negative impact on training. What's more, the difference of faces is only in the facial features, skin color and hair, while the overall structure of the faces is the same. However, for different types of garments, the overall shape of them is diverse. When our team applied StarGAN to garment images, we found several defects from the experimental results:

1. The slow convergence and instability of the loss value of the model results in slow image formation. This is due to the defects in the down sampling number of StarGAN generator and the its failure to make full use of the low-level information.
2. In the garment image generation, StarGAN reconstruction loss finally converges to a relatively large value, which results in the generated image not preserving the feature of the input image well. As a result, there is a huge difference between the texture of the generated image and that of the input image.

Therefore, based on StarGAN, this paper proposes GD-StarGAN, a multi-domain image-to-image translation model for garment design. It improves the convergence speed and stability of loss function while improving the accuracy and quality of the generated images. The following are the contributions of this paper:

1. This paper proposes a garment image-to-image translation model that can save time and energy for fashion designers.
2. This paper replaces the original residual network of StarGAN generators with U-net [7], a fully convolution network, and proves that this structure can improve the convergence speed and stability of the loss function and improve the image forming rate.
3. In this paper, the reconstruction loss function of StarGAN is improved and changed to a loss function more suitable for image-to-image translation in multiple domains and renders the texture of garment images generated by the network closer to the input texture image.

## Related work

### A, Generative Adversarial Network

The Generative Adversarial Network (GAN) consists of two networks, a discriminant network and a generative network. These are referred to as the discriminator and the generator, respectively. The generator tries to generate the same data as the real data, while the discriminator tries to improve its discriminant ability and suppress the generator. After many confrontations, the generator can eventually generate data similar to real data. Initial GAN generation capacity is still relatively weak. In recent years, many authors [8], [9], [10] have greatly improved the effectiveness of GAN.

### B, Conditional GAN

CGAN refers to the addition of conditions in the generative adversarial net. The role of conditions is to supervise the generative adversarial net so that the networks can generate specific objects under certain conditions. For example, class or attribute labels [3], [6], [11], [12], text [13], [14], and images [3], [15] can all serve as conditional information for GAN.

### C, Image-to-image GAN

Image-to-image translation GAN has made great progress in recent years. Pixl2Pix is an image-to-image translation model based on paired data. In order to solve the dependence of paired data, researchers proposed two two-domain image-to-image translation models, DiscoGAN and CycleGAN, which do not need paired data. SF-GAN [16] combines a geometry synthesizer and an appearance synthesizer to achieve synthesis realism in both geometry and appearance spaces. StarGAN is a multi-domain image-to-image translation model using only one generator and one discriminator, which is also the basic model of this paper. FUNIT [17] is a few-shot, unsupervised image-to-image translation algorithm.

### D, GAN in fashion design

Attribute-GAN [18] is a garment-matching image-to-image translation model based on CGAN. FashionGAN [19] proposes a garment design model for image-to-image translation from sketch to image. Inputting the required fabric image and fashion sketch allows the generation of an image based on the color of the sketch fabric. FashionGAN has the same purpose as GD-StarGAN. It generates images with specified textures and shapes. Unlike with FashionGAN, GD-StarGAN is only necessary to input textures and labels to generate garment images corresponding to the shape of labels, and texture images can be almost any images.

## Architecture of GD-StarGAN

This section will introduce StarGAN and U-net, as well as GD-StarGAN.

### A, StarGAN

StarGAN uses a discriminator, an auxiliary classifier [11], and a loss of cycle consistency loss (reconstruction loss) [4], [5] to train the generator to achieve multi-domain image translation. And auxiliary classifier means that both generator and discriminator use labeled data to train and utilize discriminator to reconstruct label information. Here, we assume that D is the discriminator, G is the generator, x is the input image, c is the target domain label from the label library, and *c*′ is the input image label. *c*′ and *c* are one-hot labels.Each label is represented as a binary vector, with zero values except for the label's index of one.

#### A.a, Model workflow

**Fig 1:**
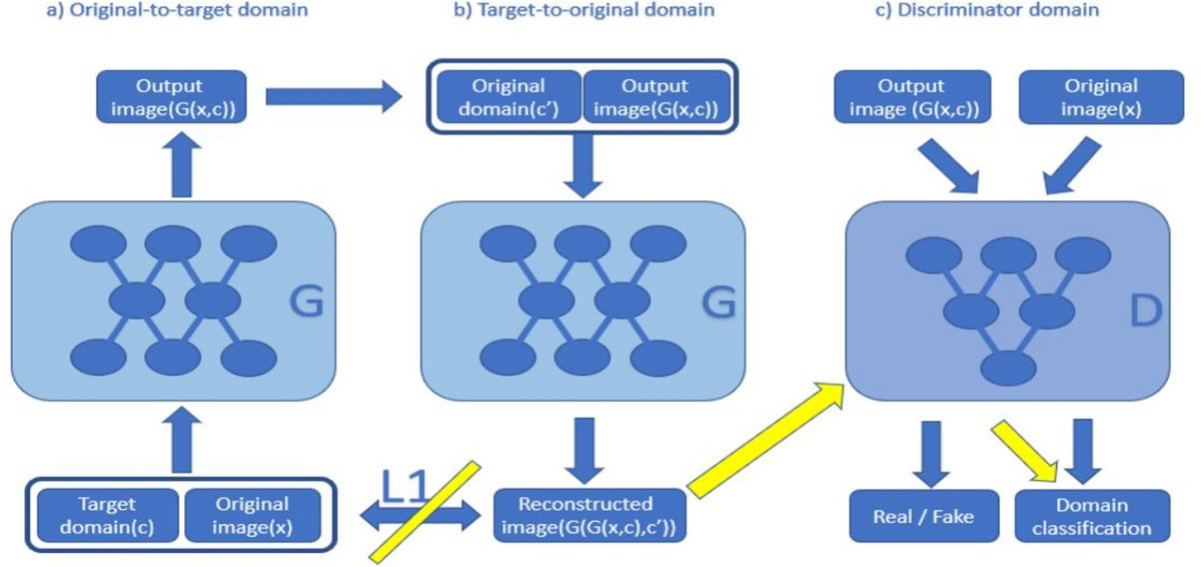
Model diagrams of StarGAN and GD-StarGAN. The yellow arrows and diagonals are our modifications to the StarGAN loss function. The reconstructed image is input into the discriminator, and the discriminator classifies it into the domain of the input image instead of using L1 loss between the reconstructed image and the input image.

As shown in Fig 1, this paper divides the whole model into three parts: original-to-target domain, target-to-original domain, and discriminator domain.

Original-to-target domain: The input image x is connected to the label c and fed into the generator to produce G (x, c).

Target-to-original domain: The output image G (x, c) is connected to the original image label c ' and is input to the same generator. Then the generator outputs the reconstructed image G (G (x, c), c '), and uses L1 loss between G (G (x, c), c ') and x.

Discriminator domain: The input to the discriminator is x and G (x, c). For x, the discriminator identifies it as real and classifies it as original image domain *c*′. For G (x, c), the discriminator identifies it as fake and classifies it as target domain *c*.

#### A.b, Loss function

$$L_{adv}=E_{x}[\log\;D(x)]+E_{x,c}[\log\left(1-D(G(x,c)\right)]$$ (1)

$$L_{cls}^{r}=E_{x,c^{\prime }}[-\log\;D_{cls}(c^{\prime }|x)]$$ (2)

$$L_{cls}^{f}=E_{x,c}[-\log\;D_{cls}(c|G(x,c)]$$ (3)

$$L_{rec}=E_{x,c,c^{\prime }}[{\left\|x-G(G(x,c),c^{\prime })\right\|}_{1}]$$ (4)

$$L_{D}=-L_{adv}+\lambda_{cls}L_{cls}^{r}$$ (5)

$$L_{G}=L_{adv}+\lambda_{cls}L_{cls}^{f}+\lambda_{rec}L_{rec}$$ (6)

The loss function of StarGAN consists of four components, which are adversarial loss (Eq 1), reconstruction loss (Eq 4) and two domain classification losses (Eq 2 and Eq 3). Eq 5 is composed of Eq 1 and Eq 2 while Eq 6 is composed of Eq 1, Eq 3 and Eq 4. They optimize discriminator D and generator G, respectively. *λ*_*cls*_ and *λ*_*rec*_ are two hyperparameters representing the importance of the corresponding loss functions. This paper sets them both to 10.

Adversarial loss: The purpose of adversarial loss is to render the discriminator unable to distinguish the generated image from the real mage. The discriminator strives to maximize it to enhance its ability to determine whether the generated image is false. On the contrary, the generator minimizes adversarial loss to render the discriminator unable to distinguish whether the generated image is true or false. Through this adversarial relationship, G’s generating ability is enhanced.

Domain classification loss: StarGAN uses an auxiliary classifier [11] to achieve multi-domain image-to-image translation of the input images. Eq 2 is used to optimize the discriminator, and Eq 3 is used to optimize the generator. By minimizing this loss function, discriminator D can learn to classify the input images x into their corresponding domains. Generator G can generate images that are classified into target domains by D.

Reconstruction loss: Reducing the value of Eq 1, Eq 2 and Eq 3 does not guarantee that the generated image will retain the characteristics of the input image. StarGAN uses L1 loss to achieve cycle consistency, which generates features in the target domain while retaining some features of the source image.

### B, U-net

The U-net network is based on fully convolutional networks (FCN) [20]. Similar to the structure of the FCN network, it can be divided into two stages: down-sampling and up-sampling. The difference is that U-net uses skip connection [7] to connect the lower sampling layer with the upper sampling layer hence that the features extracted from the down-sampling layer are transmitted directly to the up-sampling layer, which plays a significant role in image segmentation [21], [22],[23],[24].

### C, GD-StarGAN

#### C.a, Generator with skip-connect

**Fig 2:**
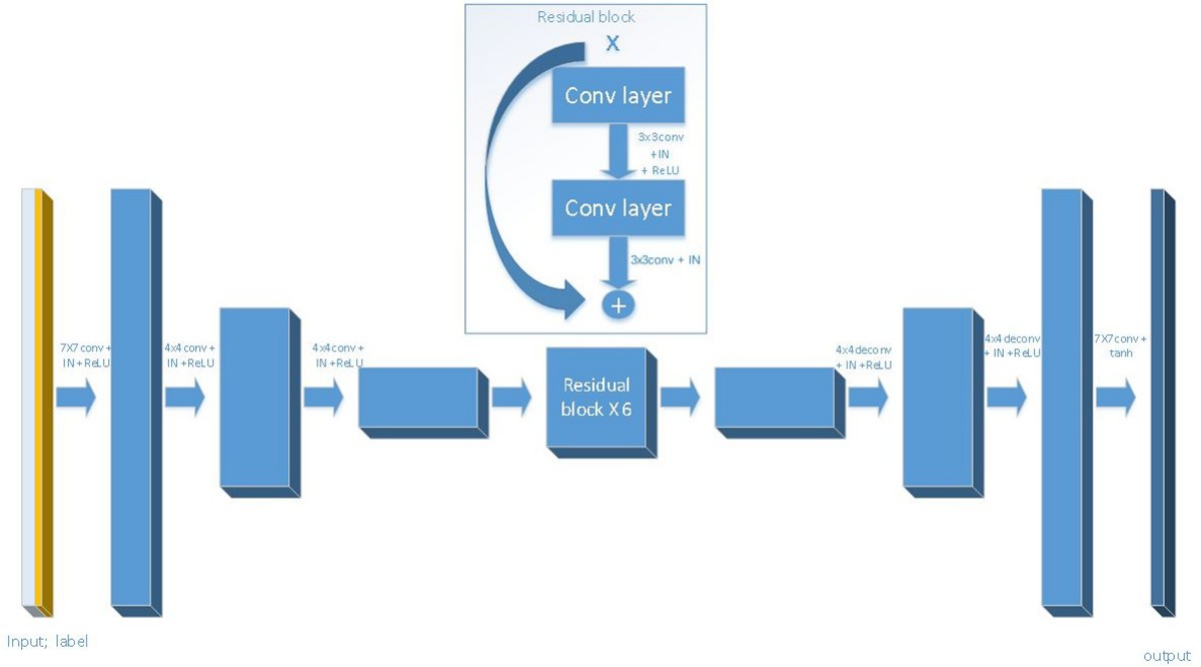
Structure of StarGAN generator. Conv denotes convolution. Deconv denotes deconvolution. The ReLU and tanh as activation function and IN denotes instance normalization.

**Fig 3:**
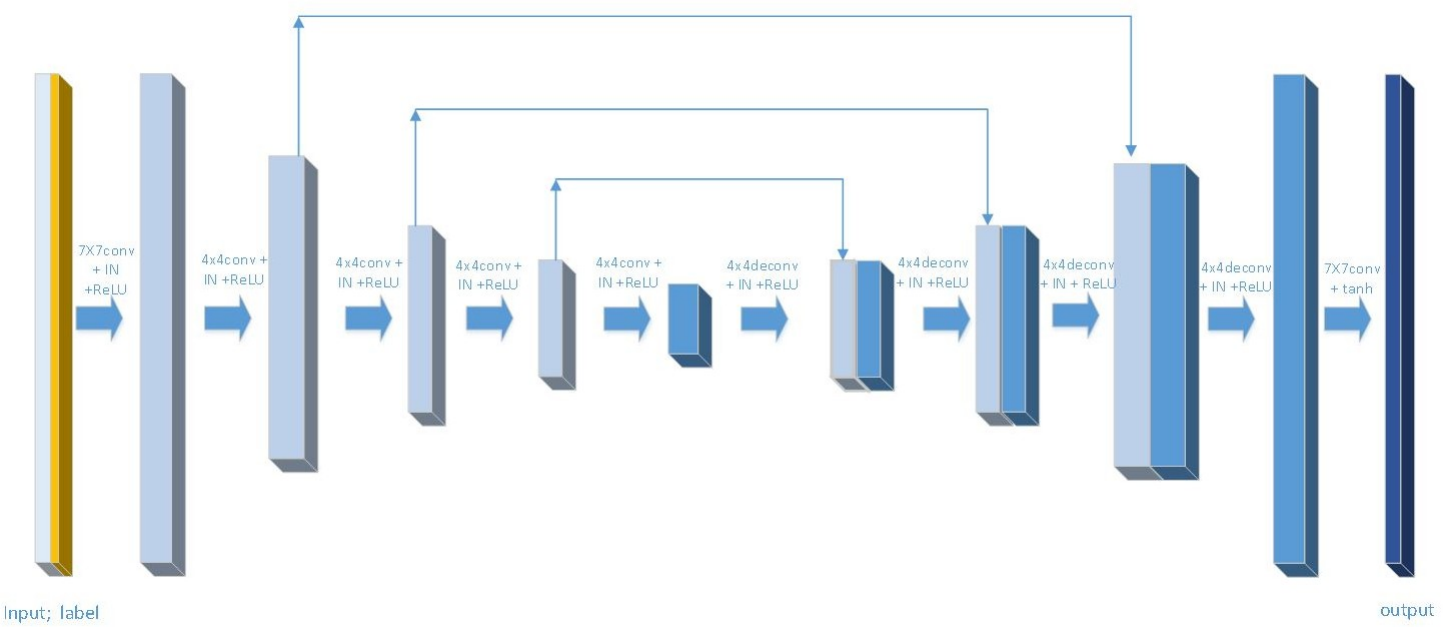
Structure of GD-StarGAN generator. The meaning of the symbol is the same as in Fig 2.

For StarGAN, as shown in Fig 2, the generation process is divided into three stages. In the first stage, the input images pass through a convolution layer and then are down sampled twice. In the second stage, the feature maps pass through six residual blocks. In the third stage, two up sampling layers and one convolution layer are used to obtain the output image. For GD-StarGAN, as shown in Fig 3, the generation process is divided into two stages. In the first stage, the input image passes through a convolution layer and is down sampled four times. In the second stage, the feature map is up sampled four times and then processed through a convolution layer to obtain the generated image. The first, second, and third down sampled feature maps are skip-connected to the third, second, and first layers of the up sampled feature maps, respectively.

For the generator, the more times of down-sampling the image, the more advanced the feature contained in the feature image. StarGAN only sampled the image twice, which means that the features of the image obtained by its down sampling are relatively low-level. Hence information contained in the feature image is relatively scattered, without integrity of the input image. The changeable scattered information makes model training unstable. Therefore, our team increased the number of samples to four.

On the other hand, one of the defining features of image-to-image conversion problems is that they map high-resolution input to high-resolution output, the input and output on the surface appearance are different, but both have the same underlying structure. That is to say the low-level information of the down sampling process and the up sampling process can be shared. Down sampling and the up sampling structure of the generator is symmetric, hence it is better to transfer the low-level information on the lower sampling process directly to the upper sampling process. To achieve this, we added skip-connect to the generator, namely using the U-net structure.

#### C.b, Improvement of loss function

A defect was found in the results of StarGAN and StarGAN with U-net; they can generate the corresponding shape of the target image but cannot maintain the same texture as the input image. In StarGAN, the authors used L1 loss to achieve cycle consistency, while this paper uses domain classification loss instead of L1 loss. As shown in Fig 1, the yellow arrows and diagonals are the modifications to the StarGAN loss function. The reconstructed image is input into the discriminator, and the discriminator classifies it into the domain of the input image instead of using the L1 loss between the reconstructed image and the input image. In short, this paper made the following changes to Eq 4:

$$L_{rec}=E_{x,c,c^{\prime }}[-\log\;D_{cls}(c\prime |(G(G(x,c),c^{\prime })]$$ (7)

Adversarial training can, in theory, learn mappings inputs that produce outputs identically distributed as target domains respectively. However, with large enough capacity, a network can map the same set of input images to any random permutation of images in the target domain, where any of the learned mappings can induce an output distribution that matches the target distribution. Thus, adversarial losses alone cannot guarantee that the learned function can map an individual input to a desired output. To further reduce the space of possible mapping functions, hence the learned mapping functions should be cycle consistency.[4] In CycleGAN, the generated input and output are fixed domains, while in StarGAN, the input and output of the same generator are constantly variational domains. However, the reconstruction loss of StarGAN generator are the same as that of CycleGAN, which is L1 Loss. L1 loss is for two domains and is not suitable for multi-domain image-to-image translation. Thus, the domain classification loss is adopted in this paper to achieve cyclic consistency. It is more suitable for multi-domain image conversion because it is a classification oriented to multiple domains.

Like with StarGAN, in order to generate high-quality images, this paper uses Wasserstein GAN [8] with a gradient penalty [9] loss function instead of Eq 1:

$$L_{adv}=E_{x}[D(x)]-E_{x,c}[D(G(x,c))]-\lambda_{gp}E_{\hat{x}}[{\left({\left\|\nabla_{\hat{x}}D(\hat{x})\right\|}_{2}-1\right)}^{2}]$$ (8)

In Eq 8, $\hat{x}$ is sampled randomly from the line between the distribution of generated image and the input image. *λ*_*gp*_ is a hyperparameter, and this paper sets it to 10.

## Experiments

This section will first describe the details of the experiments and the dataset used. It will then compare the training loss of StarGAN and StarGAN with U-net and the images they generated during several training periods. This proves that U-net can accelerate the speed of image formation. Next, a comparison is made of GD-StarGAN with StarGAN as well as StarGAN with U-net in image generation, training loss and the evaluation index, and finally the experimental results, more applications of this model and our future work are reported.

### A, Training details

All models were trained using Adam [25] as optimization with β1 = 0.5 and β2 = 0.999. For data augmentation, this research flips the images horizontally with a probability of 0.5. The batch size was set to 16 for all experiments. All models were trained for 50 epochs, and the learning rates of the generator and discriminator were both 0.0001. The training time for each model was about 6 hours on a single NVIDIA GTX 1080Ti GPU.

### B, Dataset

**Fig 4:**
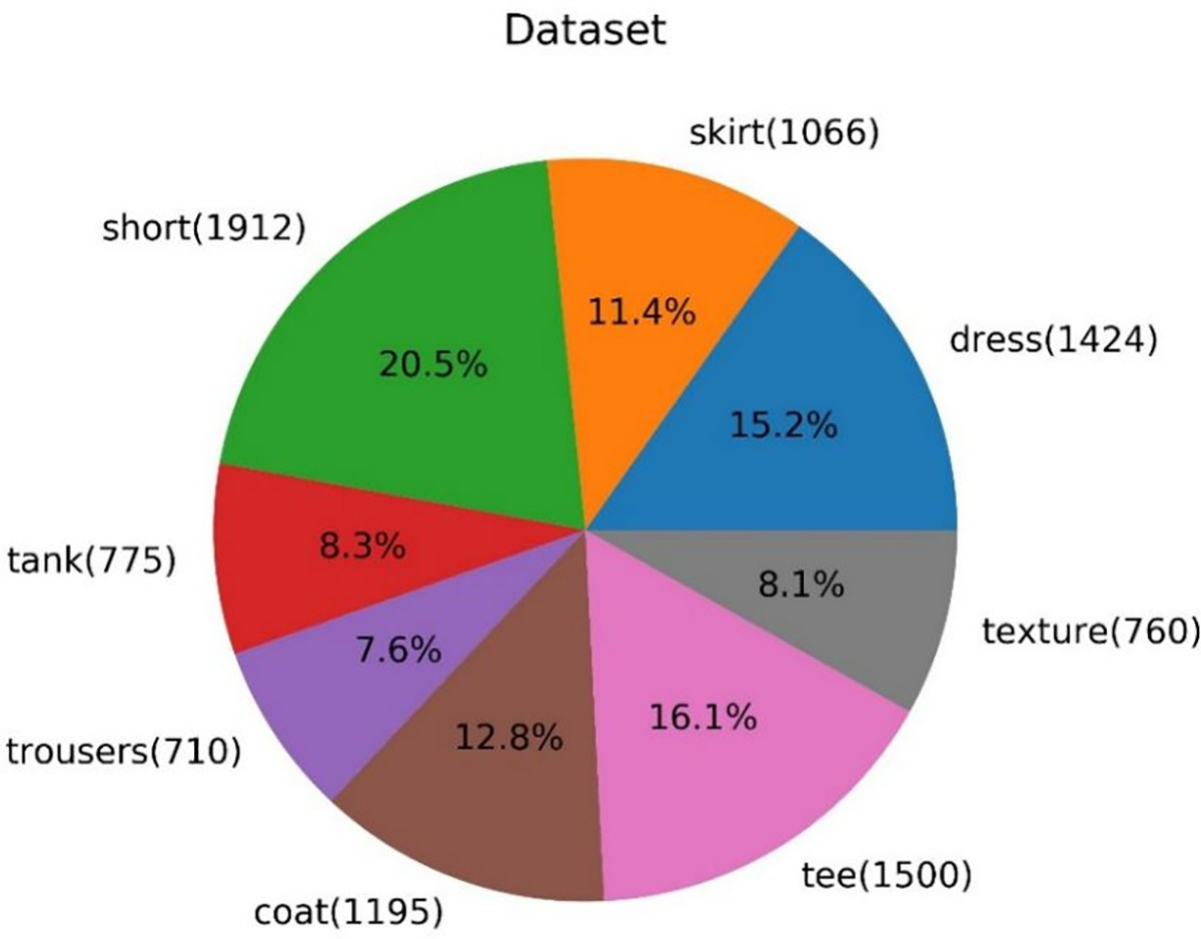
Pie chart of the proportion of all kinds of garments.

The dataset was taken from a large database of garment––Deep Fashion [26]. DeepFashion is a large-scale dataset opened by the Chinese University of Hong Kong. It includes 800,000 images with different angles, different scenes, buyer show, seller show and other images. Each image also has very rich annotation information, including 50 categories and 1000 attributes. But most of the images in this database contain models. Therefore, garment images used in this paper were classified according to their labels, and a two-class neural network was used for further classification, removing the images containing models. For texture images, web crawler, a data mining tool, was used to randomly crawl images from the web and paste their resized, rectangular shapes on a white background. In this experiment, the dataset contains 9342 images, all of which are 128*128. Fig 4 shows the proportion, type, and number of images in the dataset.

### C, Comparison

#### C.a, StarGAN and StarGAN with U-net

**Fig 5:**
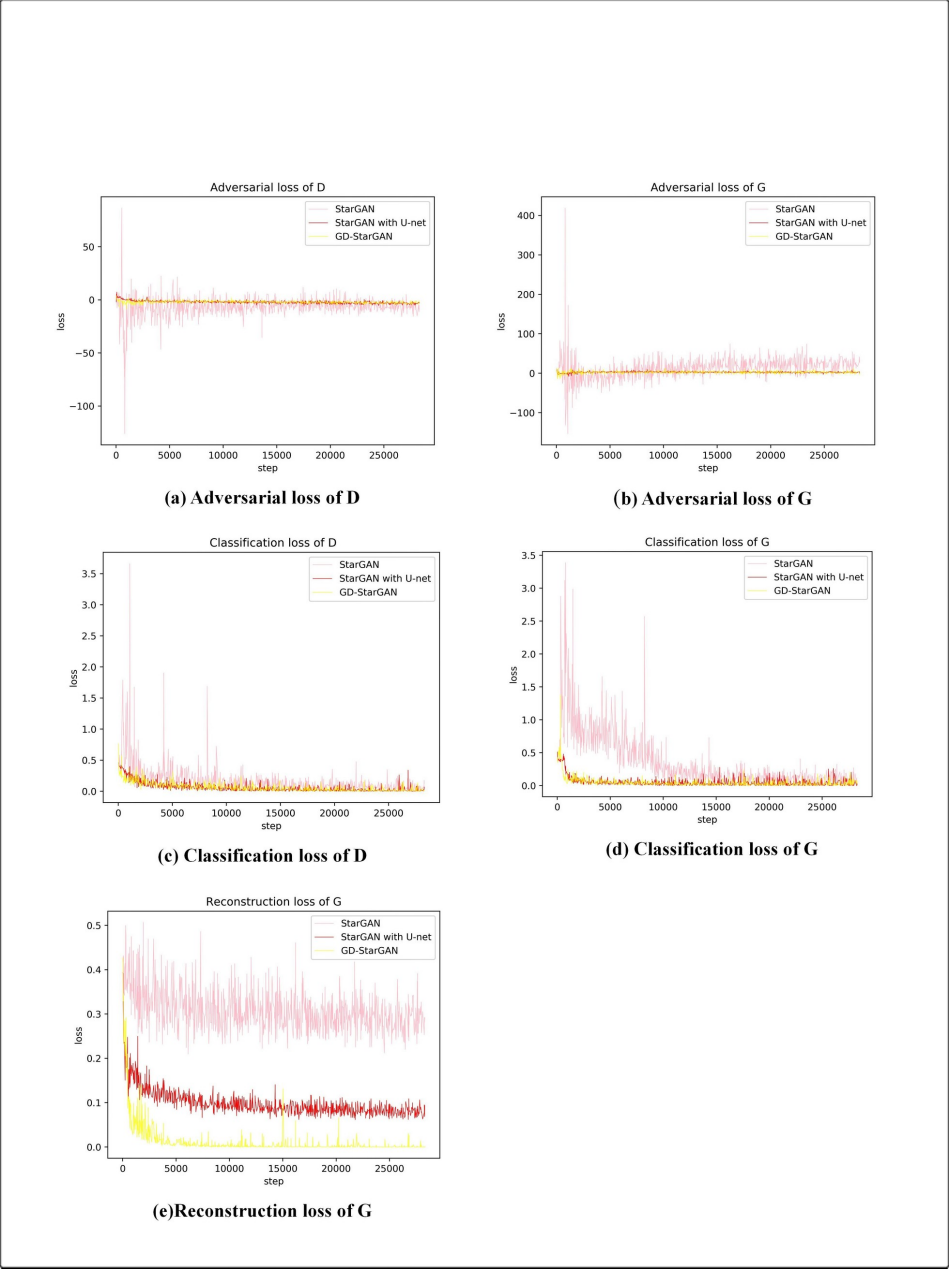
Training losses of StarGAN, StarGAN with U-net and GD-StarGAN.

As shown in Fig 5, it can be seen that the loss function tends to a stable value faster when U-net is applied, that is, the convergence speed of the loss function is accelerated. This is because the generator network of StarGAN is relatively deep, which leads to the fact that in the early training period, the gradient propagation speed is suppressed, so that the convergence speed of loss function is also slow. Additionally, U-net reduces the vibration range of the StarGAN training loss.

**Fig 6:**
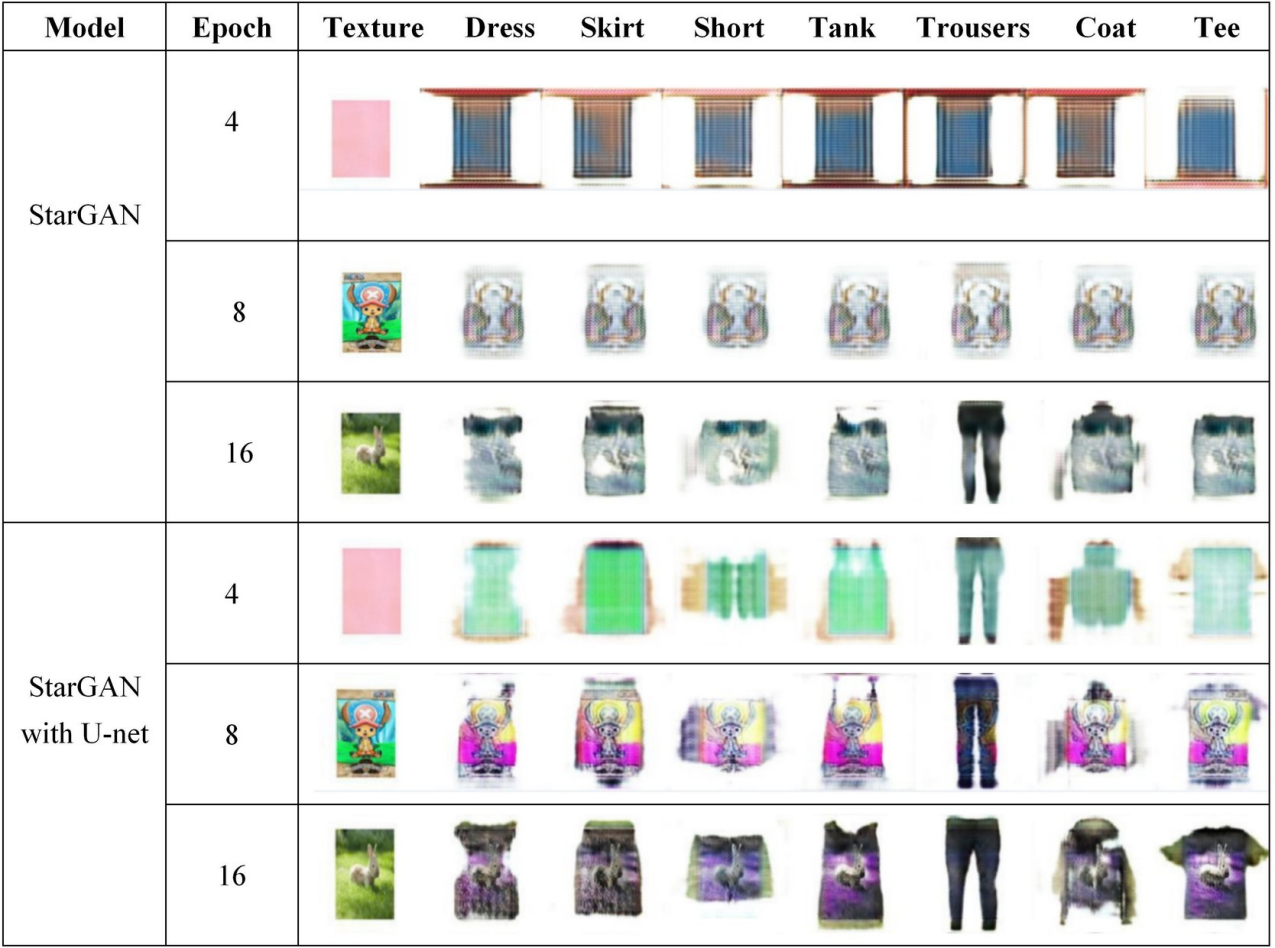
Images generated by several epochs during StarGAN and StarGAN with U-net training process.

The loss value of StarGAN with u-net is more stable and converges faster, which improves the forming speed of the image. Fig 6 shows the images generated by StarGAN and StarGAN with U-net in epochs 4, 8, and 16. It can be seen that StarGAN with U-net images are slightly better in shape and texture. This means that U-net accelerates the image formation.

#### C.b, GD-StarGAN with others

**Fig 7:**
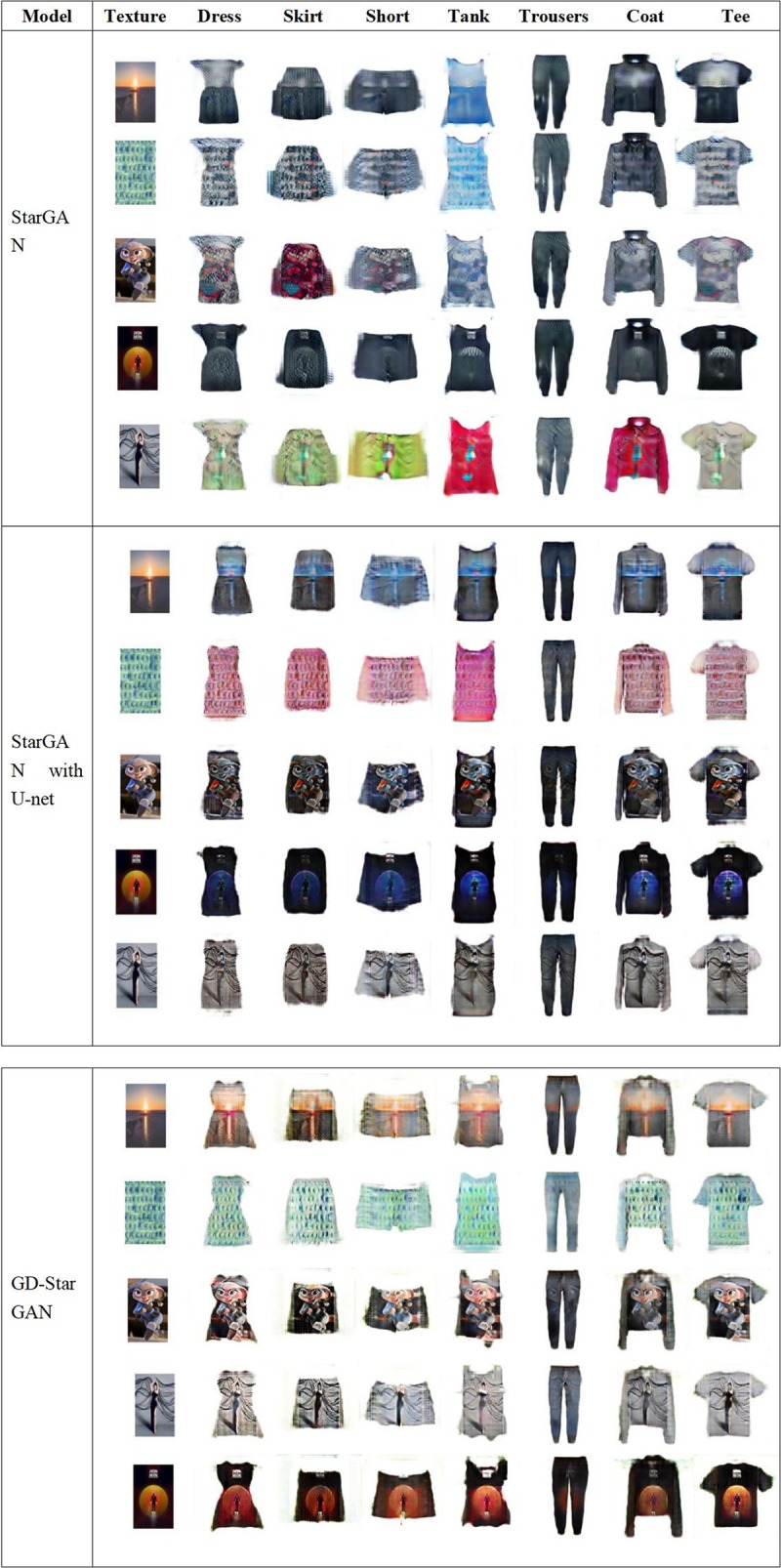
Results of StarGAN, StarGAN with U-net, and GD-StarGAN.

Fig 7 shows that although both StarGAN and StargGAN with U-net can generate garment images with corresponding shapes, the textures of the generated images differ greatly from that of the input texture images in color. GD-StarGAN differs from them in that it not only generates corresponding types of garment images but also generates garment textures close to the input texture images.

As shown in Fig 5, all the loss functions of GD-StarGAN tend to stabilize more quickly. In particular, the reconstruction loss of GD-StarGAN were much smaller than that of the others and it has the smallest overall vibration.

The inception score is an important index to evaluate the performance of GAN models. [27], [28]. The higher inception score means high quality and diversity. As can be seen from Table 1, the average inception score and the minimum inception score of GD-StarGAN is the highest of the three models. What’s more, the standard deviation of GD-StarGAN is the smallest, which means that GD-StarGAN is most steady.

**Table 1:** Inception scores for all models.

Model	Inception score
StarGAN	5.028 ± 0.180
StarGAN with U-net	5.508 ± 0.204
GD-StarGAN	5.593 ± 0.107

### D, More applications

**Fig 8:**
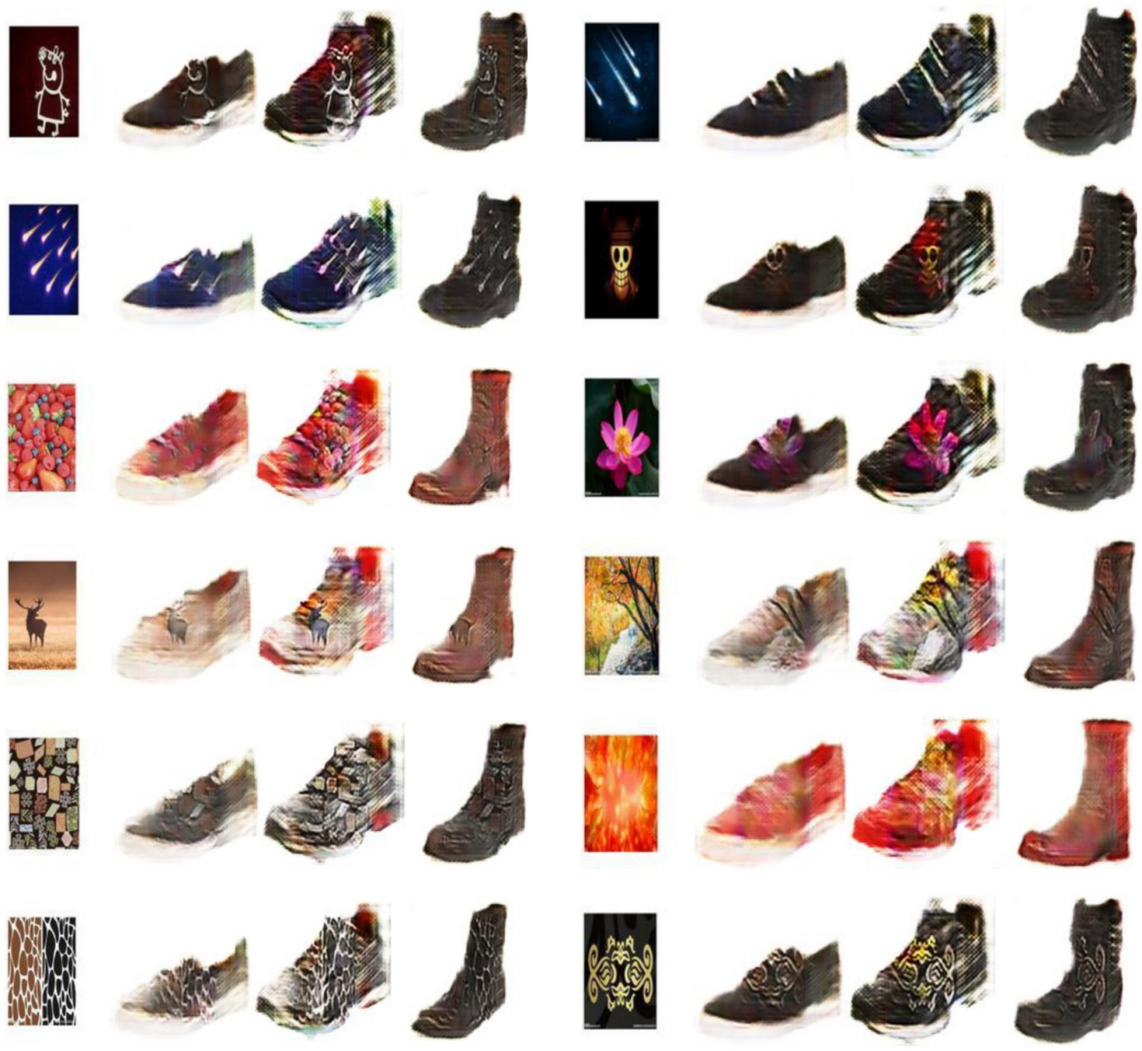
Examples of more applications.

In order to verify GD-StarGAN’s generalization, we also retrain it on shoes dataset provided by [2]. Fig 8 gives some examples of test cases. Compared with the difference between the garment images and the texture images, the difference between the shoes images and the texture images is even greater. This means that it's harder to train. But as shown in Fig 8, GD-StarGAN is still able to produce images of the shoes with the appropriate texture.

### E, Limitation and future work

**Fig 9:**
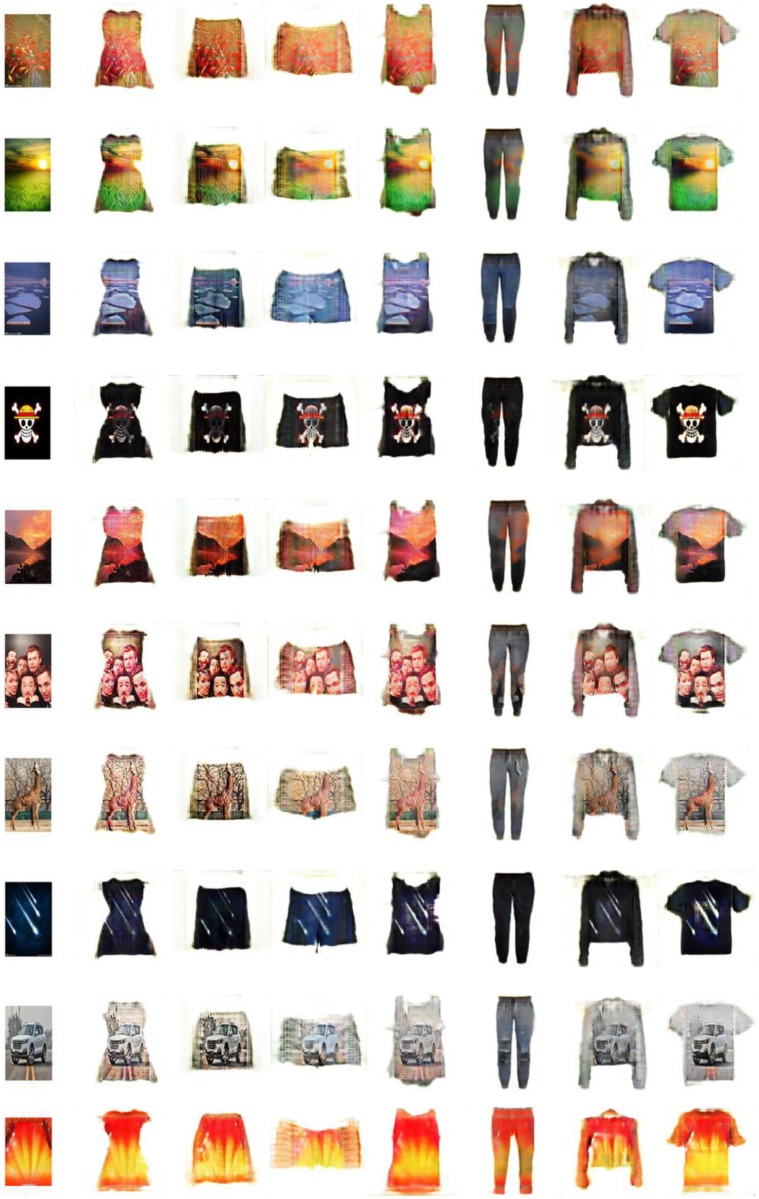
More experimental results.

Fig 9 displays more generated garment images. Although our model can generate garment images corresponding to the input texture, the obtained images are not perfect enough:(1) The edges of the garments in the obtained images are not clear enough. (2) The texture of the generated images cannot have the same clarity as that of the input texture images.

**Fig 10:**
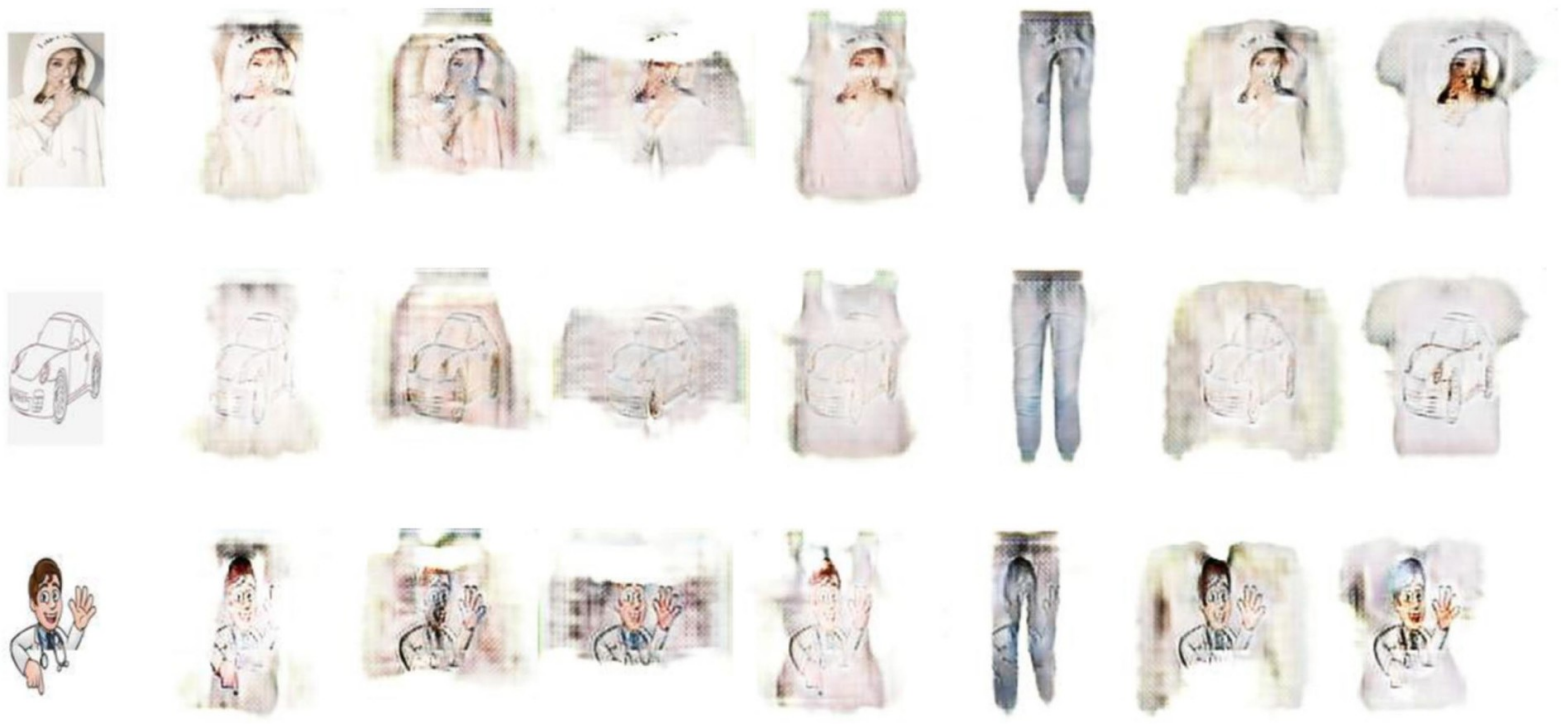
Examples of some failed results.

Fig 10 shows some of the failed samples. They are of poor quality because the input images are mostly white. And our model is based on the memorability of the shape of the training data. When the input image is mostly white, the model will take the white part of them as the background, which causes the model to fail to perform its functions.

Our future work is to develop a new model that can enhance the edges of garment images while keeping the texture clear.

## Conclusion

This paper, based on StarGAN, proposes an image-to-image translation model for garment design. Users only need to input a texture image and a corresponding garment category label, and they can automatically generate a corresponding garment image. The StarGAN generator structure and loss function were improved, and it was verified that the garment image generated by the model was better than the garment image generated by StarGAN, which solves the problem that the image generated by StarGAN has a low forming rate and texture that cannot match the input texture images. Experiments show that the model achieves better results in garment design.

## Supporting information

S1 Appendix [29], [30], [31]. (DOCX)
