# Supplementary material for: GD-StarGAN: Multi-domain image-to-image translation in garment design
Source: PLoS One. 2020 Apr 21;15(4):e0231719. doi: 10.1371/journal.pone.0231719 (PMC7173925; doi:10.1371/journal.pone.0231719)
Supplement: S1 Appendix — (DOCX) [file pone.0231719.s001.docx]

**Appendix**

The network architectures of GD-StarGAN are shown in Table 2 and Table 3. Conv denotes convolution. Deconv denotes deconvolution. The numbers in brackets in the table represent the length, width, and channels of the image. For the generator network, this paper uses Rectified Linear Units (ReLU) [29] as its activation function and applies instance normalization [30]. For the discriminator network, this paper uses Leaky ReLU [31] as its activation function. Other relevant notations include $n_{d}$: domain number, N: the number of output channels, K: kernel size, S: stride size, P: padding size, *2: skip connection.

**Table 2 Generator network architecture**

| **Part** | **Input → Output Shape** | | **Layer information** |
| --- | --- | --- | --- |
|  | (128, 128, 3) → (128, 128, 64) | | conv-(N64, K7x7, S1, P3), IN, ReLU |
|  | (128, 128, 64) → (64, 64, 128) | | conv-(N128, K4x4, S2, P1), IN, ReLU |
| Down-sample | | (64,64,128) → (32,32,256) | conv-(N256, K4x4, S2, P1), IN, ReLU |
|  | (32,32,256) → (16,16,256) | | conv-(N256, K4x4, S2, P1), IN, ReLU |
|  | (16,16,256) → (8,8,256) | | conv-(N256, K4x4, S2, P1), IN, ReLU |
|  | (8,8,256*2) → (16,16,256) | | deconv-(N256, K4x4, S2, P1), IN, ReLU |
|  | (16,16,256*2) → (32,32,256) | | deconv-(N256, K4x4, S2, P1), IN, ReLU |
| Up-sample | (32,32,256*2) → (64,64,128) | | deconv-(N128, K4x4, S2, P1), IN, ReLU |
|  | (64,64,128) → (128,128,64) | | deconv-(N64, K4x4, S2, P1), IN, ReLU |
|  | (128,128,64) → (128,128,3) | | deconv-(N256, K7x7, S1, P3), tanh |

| **Table 3 Discriminator network architecture** | | | |
| --- | --- | --- | --- |
| **Layer** | **Input → Output Shape** | **Layer Information** | |
| Input Layer | (128, 128, 3) → (64, 64, 64) | | conv-(N64, K4x4, S2, P1), Leaky ReLU |
| Hidden Layer | (64, 64,64) → (32,32,128) | conv-(N128, K4x4, S2, P1), Leaky ReLU | |
| Hidden Layer | (32,32,128) → (16,16,256) | conv-(N256, K4x4, S2, P1), Leaky ReLU | |
| Hidden Layer | (16,16,256) → (8,8,512) | conv-(N512, K4x4, S2, P1), Leaky ReLU | |
| Hidden Layer | (8,8,1024) → (4,4,1024) | conv-(N1024, K4x4, S2, P1), Leaky ReLU | |
| Hidden Layer | (4,4,1024) → (2,2,2024) | conv-(N2048, K4x4, S2, P1), Leaky ReLU | |
| Output Layer1 | (2,2,2024) → (1,1,1) | conv-(N1, K4x4, S1, P1) | |
| Output Layer2 | (2,2,2024) → (1,1, $n_{d}$) | conv-(N$n_{d}$, K2x2, S1, P0) | |
